# Editorial: Debates in clinical management in pediatric endocrinology, volume II

**DOI:** 10.3389/fendo.2023.1270794

**Published:** 2023-08-21

**Authors:** Gianpaolo De Filippo, Barbara Predieri, Maurizio Delvecchio

**Affiliations:** ^1^ Assistance Publique - Hôpitaux de Paris, Service d’Endocrinologie et Diabétologie Pédiatrique, Hôpital Robert Debré, Paris, France; ^2^ Pediatric Unit, Department of Medical and Surgical Sciences of The Mother, Children and Adults, University of Modena and Reggio Emilia, Modena, Italy; ^3^ Metabolic and Genetic Disorders, “Giovanni XXIII” Children’s Hospital, Bari, Italy

**Keywords:** Wolfram (DIDMOAD) syndrome, NASH, NAFLD, childhood obesity, type 1 diabetes mellitus, pediatric endocrine disorder, thyroid cancer, iodine supplementation

Despite the progress in diagnosis, management and treatment of pediatric endocrine disorders, some questions such as “And now? How can I treat this patient? What is the best therapeutic approach in this situation?” still come across the mind of pediatricians during daily clinical practice. This Research Topic is the Volume II of the previous Research Topic “*Debates in clinical management in pediatric endocrinology*” (1), which included 6 papers. This volume includes other 6 manuscripts (2 reviews and 4 original papers).

Some aspects of widespread diseases, like obesity and type 1 diabetes mellitus, are covered, as well as unsolved concerns of everyday practice (i.e. diagnosis of persistent glucocorticoid-induced adrenal-insufficiency). Furthermore, this volume contains an interesting observation dealing with phenotypical heterogeneity of Wolfram syndrome and last but not least, a review on risk resulting from nuclear disaster, which brings us back to daily international current events.

Non-alcoholic fatty liver disease (NAFLD) represents the biggest non-communicable liver disease and the leading indication for liver transplantation in the United States. A meta-analysis of 8.5 million adults estimated the global prevalence of NAFLD at 25% (2), affecting 45% of all Latinos, 33% of all Caucasians and 24% of all Africans Americans, both fat and thin. Prevalence in pediatric age is estimated between 3 and 10%, according to different diagnostic criteria (3). Considering this disease was not even described until 1980, the increase in prevalence is astounding. Most of the people with NAFLD have no symptoms and do not even know they have it. The majority of them will suffer no ill effects, but 5% of them will go on to develop NASH, with inflammation and scarring of the liver. Among them, 25% will develop cirrhosis. An unhealthy dietary pattern such as saturated fats, carbohydrates, soft drinks, red meat and ultra-processed foods are linked to NAFLD. Barbieri et al. in this Research Topic present a complete review on NAFLD in youth with obesity, analyzing the physiopathology of the disease. While there are certain genetic predispositions, accounting for the higher prevalence in Latinos, the large number of elements apart from genetic factors leading to NAFLD phenotype (i.e. obesity, insulin-resistance, microbiota), largely influenced by dietary habits, show the relevance of the so-called “obesogenic environment” (4).

Still about the topic of childhood obesity and related diseases, the interesting article by Casale et al. highlights how obesity and precocious puberty are more frequent in individuals with narcolepsy type 1 (NT1). This is a disorder that recognizes a clear pathophysiological basis: incretin (or orexin) deficiency secondary to autoimmune destruction of the neurons that produce it. Disruption of the orexin system induces broader disorders, impacting feeding behavior and the hypothalamic-pituitary-gonadal axis. Furthermore, the observation points out the association between sleep and the re-awakening of the neuro-reproductive axis, as reported in several previous studies (5).

Taken together, these observations could be a useful experimental model for the impact of sleep disorders on the gonadal axis, irrespective of pathologies specifically concerning sleep-wake rhythm. The topic is interesting, given the reduction in sleep time found in adolescents. A study involving 1502 adolescents aged between 10 and 17 (6) and taking into account various lifestyle determinants finds an association between poor sleep (in terms of duration and quality) and an increased metabolic and cardiovascular risk, in particular concerning impaired glucose metabolism. In this context, we can clearly see the instauration of a vicious circle involving sleep disorders, obesity and metabolic disorders. In the current context of the obesity epidemic, it’s not surprising to see how two seemingly unrelated articles can have so much in common.

Management of T1DM during schooldays may be a challenge for a lot of children. International Guidelines focus on the risk of the diabetes management at school, emphasizing the importance of collaboration and communication between the stakeholders for the purpose of creating a safe environment at school (7). Ding et al. investigate the effect of school life in 78 students with T1DM on continuous glucose monitoring metrics, on the basis of holidays and schooldays. Time in range was significantly higher during holidays than during schooldays, with a more pronounced difference during nighttime. Interestingly, nocturnal glycemic control was worse in girls than in boys. As a matter of fact, the glycemic control worsened during nighttime during schooldays.

The interesting paper by Laulhé et al. aims to evaluate the role of morning cortisol values in the prediction of glucocorticoid-induced adrenal insufficiency as compared to the low dose short synacthen test. The authors retrospectively collected data from 91 pediatric patients who underwent the dynamic test. Glucocorticoid-induced adrenal insufficiency was detected in 60% of them. A plasma cortisol level below 144 nmol/l was able to predict adrenal insufficiency with a specificity of 94%, and on the other hand, a plasma cortisol above 317 nmol/l predicted recovery of the hypothalamus-pituitary-adrenal axis, with a sensitivity of 95%. The authors suggest that morning cortisol levels can be safely used to assess recovery of the hypothalamus-pituitary-adrenal axis in these patients avoiding the dynamic test in more than 50% of them. Few studies have been run on the topic, and this paper adds useful data for clinicians.

Wolfram syndrome type 1 (WFS1) is a heterogeneous disorder, characterized by diabetes mellitus and optic atrophy. Deafness and diabetes insipidus, and other minor clinical features, may be present as well. It is well acknowledged that the pathogenic variant does not predict the phenotype of WFS1, even in the same family (8). In their original paper, Ding et al. report on three probands and elder brother with WFS1 genetically confirmed from three different families. RNA sequencing was performed on the patients, the parents with WFS1 variants, and four gender- and age-matched children living with type 1 diabetes mellitus (T1DM). All of them underwent a comprehensive clinical and radiological evaluation. The authors describe a differentially expressed genes associated with immune-related pathways. These data confirm and highlight the clinical and genetic heterogeneity in patients with the same WFS1 variant, even if in the same family.

The 2022 War in Ukraine sadly called attention for nuclear disaster and the risk of health. In this point of view, the review by Calcaterra et al. summarizes the risk for thyroid irradiation and related cancer. The intake of stable iodine was suggested to play a significant role in the prevention of childhood thyroid cancer. The saturation with table iodine inhibits the uptake of radioactive iodine. The authors discuss the benefits and the adverse effects of the prophylaxis during a nuclear accident. Evidence suggests that large doses of iodine are well tolerated without any adverse effects, but a prolonged assumption of the iodine can be dangerous. Finally, we would like to thank all the authors who published in this Research Topic. We hope that the readers will find this volume interesting and useful for clinical practice.

## Author contributions

GDF: Writing – original draft, Writing – review & editing. BP: Writing – original draft, Writing – review & editing. MD: Writing – original draft, Writing – review & editing.
